# Hydrocracking of Jatropha Oil over non-sulfided PTA-NiMo/ZSM-5 Catalyst

**DOI:** 10.1038/srep41654

**Published:** 2017-01-30

**Authors:** Xiaosong Yang, Jing Liu, Kai Fan, Long Rong

**Affiliations:** 1 Key Laboratory for Biomechanics and Mechanobiology of Ministry of Education, School of Biological Science and Medical Engineering, Beihang University, Beijing 100191, P. R. China; 2 Beijing Key Laboratory of Lignocellulosic Chemistry, College of Materials Science and Technology, Beijing Forestry University, Beijing 100083, P. R. China

## Abstract

The PTA-NiMo/ZSM-5 catalyst impregnated with phosphotungstic acid (PTA) was designed for the transformation of Jatropha oil into benzene, toluene, and xylenes (BTX) aromatics. The produced catalyst was characterized by N_2_ adsorption-desorption, powder X-ray diffraction (XRD), Fourier transform infrared (FT-IR), X-ray photoelectron spectroscopy (XPS), and the temperature-programmed desorption of ammonia (NH_3_-TPD). The catalytic performance was evaluated by gas chromatography (GC). The liquid products were 70 wt% of the feed oil, and the majority of the liquid products were BTX. The aromatization activity of the catalyst was improved by the addition of PTA and the hierarchical process. The favorable PTA amount was 20 wt% and the yield of BTX was 59 wt% at 380 °C, 3 MPa, H_2_/oil (v/v) = 1000 and LHSV = 1 h^−1^ over the PTA20-NiMo/HZ0.5 catalyst (PTA 20 wt%) without sulfurization.

The hydrocracking of vegetable oils (Jatropha oil, palm oil, algae oil, etc.) for use as bio-fuels or bio-chemicals is a promising solution due to the problem of depleting petroleum reserves as well as environmental concerns^1^,^2^,^3^. The Hydrocracking is commonly applied to convert vegetable oils into biodiesel consisted of normal alkanes and isoalkanes. In our previous work, the biodiesel was produced successfully from hydrocracking of Jatropha oil using a non-sulfide catalyst^4^,^5^,^6^. Another study reported that vegetable oils could be utilized in the production of bio-chemicals, especially aromatic compounds^7^,^8^,^9^. Aromatics, especially monocyclic aromatics such as benzene, toluene, and xylene (BTX) have industrially important applications. These compounds can be used as a lead substitute for the octane number boosting of transportation fuel, and the seal of the engine can be made to swell by aromatics to prevent gas or oil leakage^7^,^10^. For these reasons, aromatics are often added to jet fuels to guarantee its quality^11^. Therefore, it is important to find a new method to efficiently prepare bio-aromatics from vegetable oil.

Currently, conventional fuels are derived from petroleum through distillation processes, and aromatics are derived from catalytic reforming technology^11^. It was reported that ZSM-5 zeolite was suitable as a catalyst for the production of aromatics from vegetable oils^7^. Use of this catalyst for the processing of Jatropha oil resulted in an aromatic yield of 28 wt%^8^. However, this yield was still too low to effectively use.

Hierarchical zeolite preserves the zeolitic properties of its bulk analogue but exhibits greatly enhanced active-site accessibility due to a reduction in the size of the microporous domains in which the diffusion of hydrocarbons is often strongly constrained. The pore structure may prolong catalyst lifetime by enhancing the tolerance to coking during the aromatization of branched alkenes^12^,^13^,^14^,^15^,^16^,^17^. The introduction of additives such as metal or sulfur active phase is expected to promote the transfer of hydrogen atoms or help to maintain the catalysts in this form^18^,^19^,^20^. The yield of aromatics can be improved by additives^21^. Metals (Ni, Mo, Co, Fe, and Zn) can be supported on ZSM-5, and metal sulfide catalysts have been widely used for the cracking process^1^,^20^,^22^,^23^,^24^,^25^. The hydrocracking of Jatropha oil was performed by NiMo-modified hierarchical ZSM-5 to obtain the highest total aromatic yield, approximately 50 wt%, but the catalyst was presulfided^9^. Although the yield of the product can be increased by use of a presulfide process, it results in the environmental harmful consequences of sulfur dioxide emissions, corrosion, and sulfur residues.

In this work, a modified ZSM-5 catalyst was employed with the goal of increased BTX aromatic production. Heteropoly acids (HPAs), especially those with the Keggin type, such as phosphotungstic acid (PTA), possess unique properties, and the immobilization of Keggin-type HPAs on zeolite can improve its regenerability and activity to suppress the generation of outgrowths^26^,^27^. In our previous work, environmentally friendly PTA catalysts (Ni-PTA/Al_2_O_3_ and Ni-PTA/nano-hydroxyapatite) were prepared without sulfurization for use in a clean process to generate high yields of green diesel from Jatropha oil^5^,^6^. In this work, the hierarchical ZSM-5 impregnated with PTA or/and NiMo were developed for the production of aromatics and light oil from the hydrocracking of Jatropha oil without sulfidation. The performance and the possible reaction pathway of the mechanism with the prepared catalysts were also investigated.

## Methods

### Catalyst preparation and characterization

The catalysts were developed by the incipient wetness impregnation method. All used reagents were of analytical grade and were used without further purification. The MFI-type ammonium ZSM-5 zeolite (NH_4_-ZSM-5) with a SiO_2_/Al_2_O_3_ mole ratio (SAR) of 25 was supplied by the Nankai University Catalyst Co., Ltd. Ni(NO_3_)_2_·6H_2_O, (NH_4_)_6_Mo_7_O_24_·4H_2_O, H_3_PW_12_O_40_ (PTA), NH_4_NO_3_, NaOH, and HCl were purchased from the Sinopharm Chemical Reagent Co., Ltd.

A typical treatment procedure for the support was as described previously with some modifications^28^. Briefly, the NH_4_-ZSM-5 powder was calcined at 550 °C in air for 5 h to convert the powder from the ammonium form to its protonated form (HZSM-5), denoted as HZ. The HZ zeolite supports (10 g) were soaked in 100 mL of NaOH solution at different concentrations (0.2, 0.5, and 1.0 M) for 30 min at 65 °C and 600 rpm. The solution was then filtered off and thoroughly washed with deionized water. The desilicated zeolites were subsequently treated with 100 mL of 0.1 M aqueous HCl under identical conditions for 6 h to remove the alumina debris. The sample was washed with deionized water until reaching neutral pH, and then filtered, dried at 120 °C for 12 h and calcined at 550 °C for 5 h to obtain the hierarchical ZSM-5 zeolites. The obtained samples were transformed into a protonated form by ion-exchanging three times in 0.5 M NH_4_NO_3_ solution at 80 °C for 4 h, followed by the separation−calcination procedure as described above. The hierarchical samples were denoted as HZ0.2, HZ0.5, HZ1.0, respectively, corresponding to the NaOH concentration.

Ni (4.0 wt%) and Mo (12 wt%) were loaded onto the hierarchical ZSM-5 zeolites by incipient wetness impregnation with an aqueous solution containing Ni(NO_3_)_2_ and (NH_4_)_6_Mo_7_O_24_^9^. Impregnated samples were dried at 120 °C for 12 h and calcined at 500 °C for 5 h, denoted as NiMo/HZ.H_3_PW_12_O_40_ (PTA) was also loaded on the NiMo/HZ zeolites by impregnation with an aqueous solution containing 5 to 45 wt% PTA. Impregnated samples were dried at 120 °C for 3 h and calcined at 500 °C for 5 h, denoted as PTA(x)-NiMo/HZ (where × indicates the PTA content, from 5 to 45 wt%)^6^.

X-ray diffraction (XRD) was performed to determine the structural properties of the samples using a Rigaku D/MAX-2500 X-ray diffractometer with Cu Kα (λ = 0.1541 nm) radiation at 40 kV and 40 mA. Each sample was scanned at a speed of 4° min^−1^ over a range from 5° to 80°. Analysis was carried out with Highscore plus analysis software equipped with a standard ICDD X-ray diffraction database supplied by Panalytical.

Chemical analysis of the composite was carried out using a Fourier transform infrared (FT-IR) spectrophotometer (Lambda FTIR-7600) in the range from 4000 cm^−1^ to 400 cm^−1^ at 1.5 cm^−1^ resolution, averaging 32 scans.

The Si/Al mole ratio (SAR) of the samples was determined by inductively coupled plasma optical emission spectrometry (ICP-OES) using an Optima-7000DV instrument equipped with photomultiplier tube detection.

The specific surface area, pore volume, and average pore diameter of catalysts were determined by N_2_ adsorption and desorption isotherms using the Quantachrome NOVA 2200e Surface Area and Pore Distribution Analyzer instrument at −196 °C. The samples were degassed at 300 °C for more than 6 h under high vacuum conditions. The specific surface area was calculated using the Brunauer Emmett Teller (BET) method based on the adsorption data (P/P_0_ range = 0.05–0.30). The t-plot method was used to discriminate microporosity and mesoporosity. The mesopore surface area and micropore volume were determined from the isotherms using the t-plot method. The mesopore volume and micropore diameter were determined from the isotherms using the density functional theory (DFT) method. In order to avoid the Tensile Strengthening Effect, the average mesopore diameter was determined from the isotherms using the Barrett Joyner Halenda (BJH) method.

X-ray photoelectron spectroscopy (XPS) data were obtained using an ESCALab 250 electron spectrometer from Thermo Scientific Corporation at 280 eV pass energy. The base pressure was about 6.5 × 10^−10^ mbar. The binding energies were corrected for sample charging using the C 1 s peak at 284.6 eV for adventitious carbon as a reference. The peak areas of the samples were determined by measuring the Ni 2p and Mo 3d peak areas (after linear subtraction of the background).

The acidity of catalysts was measured with a temperature programmed desorption of ammonia (NH_3_-TPD) using a chemisorption analyzer (TP-5080 Multi-functional automatic Adsorption Instrument, Tianjin Industrial Technology CO., Ltd) with a thermal conductivity detector. Prior to each TPD run, the catalyst (0.2 g) was heated to 500 °C for 30 min under N_2_ to remove all moisture. The catalyst was then cooled to 100 °C and saturated with NH_3_, and then flushed with N_2_ to remove the physically adsorbed NH_3_. Finally, desorption of NH_3_ was performed from 100 °C to 600 °C at a heating rate of 10 °C min^−1^.

### Catalytic activity measurements

The Jatropha oil was purchased from Jiangsu Donghu Bioenergy Co., Ltd, including myristic acid (0.8%), arachic acid (0.5%), linolenic acid (0.9%), palmitoleic acid (1.2%), stearic acid (7.3%), palmitic acid (14.8%), linoleic acid (36.2%) and oleic acid (38.3%).

**Figure 1:**
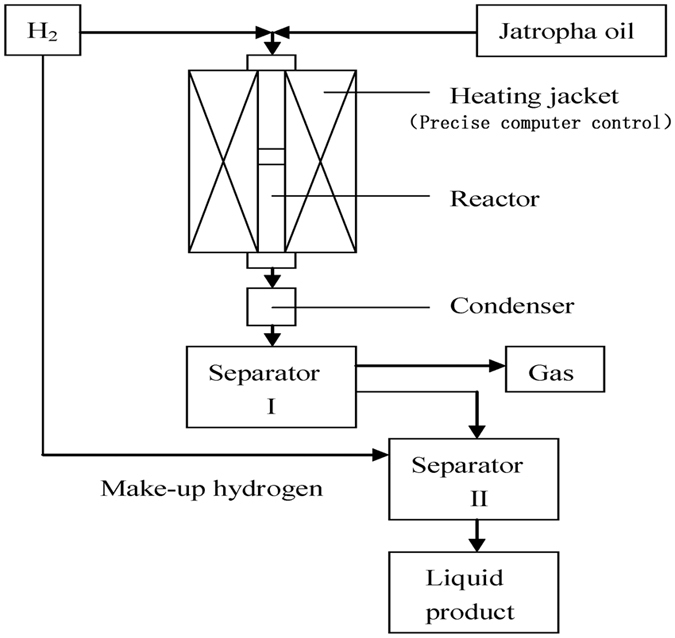
Simplified schematic diagram of the reaction apparatus (diagram was drawn by Xiaosong Yang).

All reactions were carried out in a continuous fixed-bed flow reactor (1.2 cm I.D. and 56 cm in length) equipped with an electrically heating system from Tianjin Golden Eagle Technology Co., Ltd, (JF-2). The equipment for continuous hydrocracking consisted of a feed system, heating section, tubular reactor, condensation section, storage section, and an instrumentation and control section, as schematically depicted in Fig. 1. The reaction temperature was controlled by microcomputer, and the system pressure was maintained by a backpressure regulator. Jatropha oil was delivered from a feed pot into the reactor using a high pressure pump. All gases used were UHP grade and were controlled by a separate mass flow meter. Typically, 10 g non-sulfided catalyst was loaded into the reactor and heated under H_2_ to 400 °C at a ramp rate of 5 °C min-1, and then incubated at 3 MPa for 3 h to convert the metal into its non-oxide form. The main feedstock was mixed with high pressure hydrogen and entered the reactor where the feed molecules undergo the hydrocracking reactions. The reaction conditions for the experiment were as follows: temperature 380 °C, pressure 3 MPa, LHSV 1.0 h^−1^, and H_2_ to a feed ratio of 1000 (v/v). The product exited the reactor as a mixed gas liquid phase and was cooled before it entered a high pressure low temperature separator, where the gas and liquid phases separated for analysis.

The reaction system achieved stability after 8 h. The gas products were analyzed online by a gas chromatograph (GC-900C) equipped with a thermal conductivity detector (TCD) and two columns, a TDX-01 packed column (3 m in length and 3 mm I.D.) for the determination of CO and CO_2_, and a GDX-104 packed column (3 m in length and 2 mm I.D.) for the determination of light hydrocarbons (C1–C4). The liquid products were analyzed by using a GC-900C equipped with a flameionization detector (FID) using a AT.SE-30 capillary column (30 m in length and 0.32 mm I.D.) with a film thickness of 0.5μm. The following temperature program was used: the injection port and detector temperature were set to 300 °C, the initial temperature was 50 °C for 5 min, followed by heating at 8 °C min^−1^ to 180 °C, then heating at 15 °C min^−1^ to 210 °C, and heating at 8 °C min^−1^ to 280 °C, with a 10 min hold at 280 °C. The individual products were identified using GC standards (purchased from Sigma-Aldrich, LLC). The triglycerides in the liquid product were determined by methyl esterification with GC analysis.

The conversion (C) of Jatropha oil was calculated as:


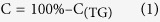


where C_(TG)_ was the concentrations of triglycerides (TG) in the liquid product.

The yield (Y) of miscellaneous hydrocarbons was calculated as:


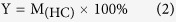


where M_(HC)_ was the mass of the corresponding hydrocarbons (HC) in the liquid product.

## Results and Discussion

### Catalyst characterization

**Figure 2:**
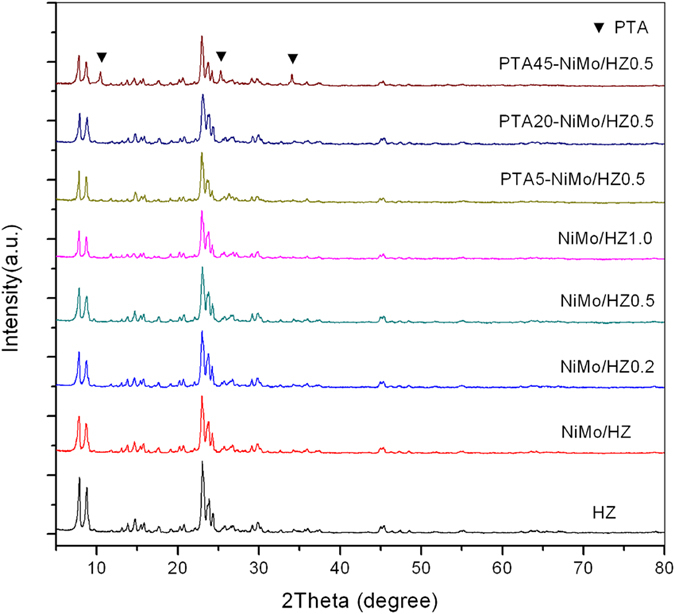
XRD patterns of the ZSM-5 catalysts.

The X-ray diffraction patterns of all freshly prepared ZSM-5 catalysts are shown in Fig. 2. The patterns exhibited the characteristic diffraction peaks at 2θ = 7.89°, 8.73°, 14.82°, 23.04°, 23.86°, and 24.26°, which were indexed to the MFI zeolitic structure (JCPDS No. 42-0024). The XRD patterns of the original ZSM-5 zeolites showed that no new phases were generated and the intrinsic lattice structure of the ZSM-5 was unchanged. Thus the fine structure of zeolites was maintained in the catalyst after the desilication and impregnation process, but the relative crystallinity decreased. This result was similar to that reported previously^9^,^29^,^30^. After being impregnated with NiMo species, no peak was observed for the Ni or Mo compounds. The absence of any metal oxide peaks in the XRD patterns indicated good metal dispersion with small particle size on the zeolite surfaces^31^,^32^. After being impregnated with PTA, the peaks observed at 2θ = 10.52°, 25.54°, and 34.02° were attributed to PTA (JCPDS No. 50-0657), and were only in the PTA45-NiMo/HZ0.5 catalyst sample. This indicated that the PTA was highly dispersed on the PTA5-NiMo/HZ0.5 and PTA20-NiMo/HZ0.5 catalysts, but the PTA was aggregated due to excessive impregnation on the PTA45-NiMo/HZ0.5 sample. In addition, there were no other diffraction peaks for the impregnated PTA catalyst samples, indicating that the Keggin structures remained intact^33^.

**Figure 3:**
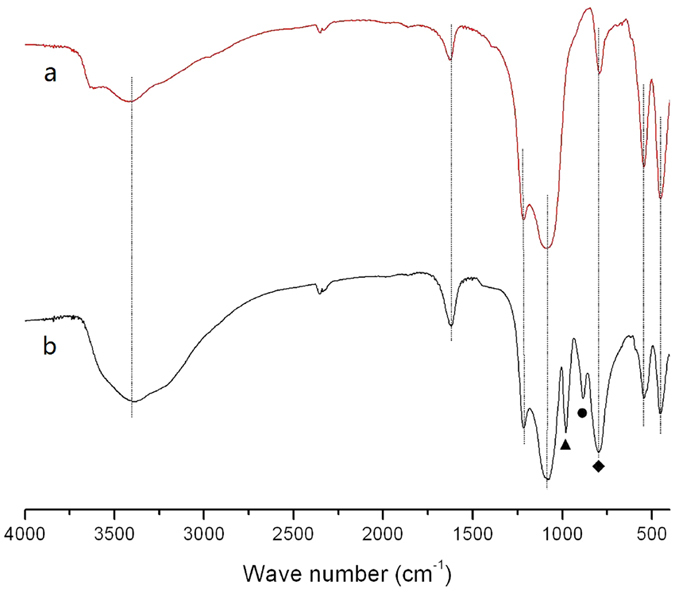
FTIR spectra of (**a**) the HZ catalyst and (**b**) the PTA20-NiMo/HZ0.5 catalyst. (▲W = O; ●W–O–W edge; ◆W–O–W corner).

The FTIR spectra of the HZ and PTA20-NiMo/HZ0.5 samples collected at room temperature are presented in Fig. 3. The characteristic bands at ν = 455, 545, and 795 cm^−1^ were associated with the stretching vibration of [AlO_4_] or [SiO_4_] groups in the HZ framework. One band at ν = 1080 cm^−1^ with a shoulder at ν = 1240 cm^−1^, were attributed to the characteristic asymmetric stretching band of a Si–O–Si bridge^34^. Overall, no obvious framework change was detected for the PTA20-NiMo/HZ0.5 catalyst, consistent with the XRD result. The bands at ν = 1637 and 3440 cm^−1^ were assigned to the bending vibration of the adsorbed water molecules, and the bands at ν = 3440 cm^−1^ were attributed to hydrogen-bonded silanol groups and water molecules adsorbed in the zeolite^34^. After PTA impregnation, the PTA20-NiMo/HZ0.5 catalyst showed new peaks at ν = 984, 892, and 811 cm^−1^ that corresponded to ν(W = O), ν(W–O–W) edge, and ν(W–O–W) corner, respectively^35^. The distance between the bands at ν = 811 and 795 cm^−1^ was too close, so they were combined smoothly into a bigger peak. Therefore, the Keggin structure of PTA was preserved in the catalyst without damage^36^.

**Figure 4:**
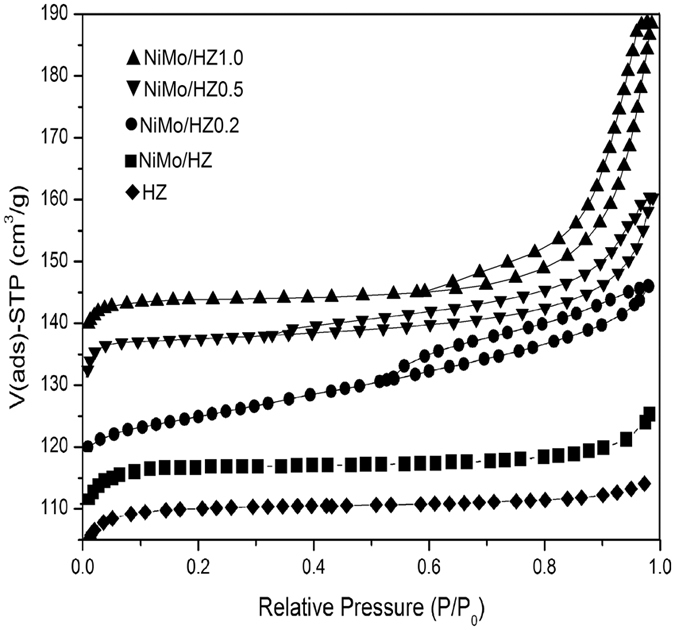
The nitrogen adsorption-desorption isotherms of the hierarchical ZSM-5 samples.

The nitrogen adsorption-desorption isotherms of the hierarchical ZSM-5 samples are shown in Fig. 4. The parent HZ zeolite presented a type I isotherm with the plateau starting at a very low relative pressure, which implied that the parent zeolite was dominated by microporous structure. After impregnation with NiMo species, the NiMo/HZ sample also presented a type I isotherm, which implied that the NiMo/HZ remained the same microporous structure. These results were consistent with the calculated micropore diameters shown in Table 1. As compared to the parent zeolite, the alkali-treated and acid-washed samples exhibited type IV isotherms typical of micro- and mesoporous materials. The isotherm with a H4-type hysteresis loop is a known fingerprint of a hierarchical porous system with size-homogeneous 1D slit channels^37^,^38^,^39^. The major differences in the isotherms of hierarchical samples compared to the parent zeolite were an increase in the N_2_ adsorption for the region 0.4 < P/P_0_ < 0.9, which was interpreted as the presence of capillary condensation in the intra-crystalline mesoporous spaces and external surface^40^. These changes should have a positive effect on the catalytic activity for the zeolite. The isotherm of the NiMo/HZ1.0 sample presented a rapid increase in absorption with P/P_0_ > 0.8, which was indicative of the substantial damage to the internal pores.

The textural properties of the various ZSM-5 samples are listed in Table 1. The Barrett Joyner Halenda (BJH) analysis of the pore size revealed mesopores in a size range of 2–20 nm. The results showed enhanced BET and mesopore surface areas for the hierarchical zeolites except the NiMo/HZ1.0 zeolite. This enhancement exhibited by the hierarchical zeolites was expected to have a positive effect on catalytic activity. The enhanced mesopore surface areas and the decreased micropore surface areas indicated that the loss in microporosity and the development of mesoporosity occurred as a result of the alkaline treatment and acid wash process. However, the development of mesoporosity occurred with micropore damage when the zeolites were treated at the higher NaOH concentration of 1.0 M, and the surface area and pore volume decreased drastically. This reduction of surface area and pore volume was expected to have a negative effect on catalytic activity. The surface area and pore volume of the NiMo/HZ zeolite catalysts decreased relative to the parent HZ zeolite, which was attributed to the action of the NiO and MO_3_ particles on the external surface to impede the access of N_2_ molecules and block the zeolite micropores. After PTA impregnation, it was clear that the surface area and pore volume decreased. The loss of specific surface area could be the result of the strong adsorption of PTA with a low surface area. Nevertheless, the decrease in surface area for low loading of PTA (5–20 wt%) was small, indicating that the majority of PTA was located outside of mesopores. However, when the PTA content was further increased to 45 wt%, the surface area and total volume were significantly decreased, which suggested that at this higher level, many micropores or mesopores of the zeolite were blocked. These results were in accordance with the XRD results.

**Figure 5:**
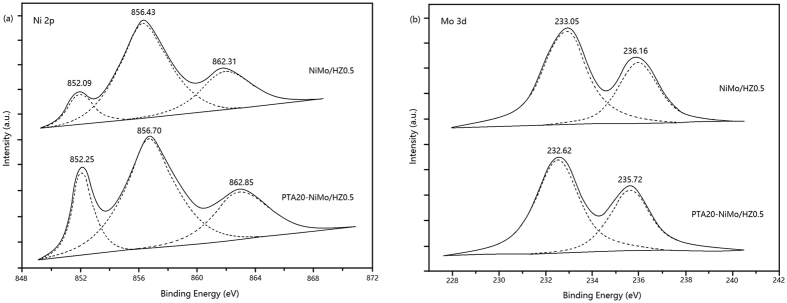
XPS spectra of (**a**) Ni 2p and (**b**) Mo 3 d levels for NiMo/HZ0.5 and PTA20-NiMo/HZ0.5 catalysts.

The XPS spectra for the NiMo/HZ0.5 and PTA20-NiMo/HZ0.5 catalysts are shown in Fig. 5. The Ni 2p binding energy is shown in Fig. 5a. The metallic Ni in the two catalysts was 852.09 eV and 852.25 eV, respectively, and the other two peaks of each sample were assigned to Ni^2+^^41^. The Mo 3d binding energy is shown in Fig. 5b. The low-binding energy (about 233 eV) was attributed to Mo^4+^ in MoO_2_, and the high-binding energy (about 236 eV) was attributed to Mo^6+^ in MoO_3_^42^. The shift of the peaks towards higher binding energy values in the Ni 2p but towards lower values in Mo 3d for the PTA20-NiMo/HZ0.5 catalyst might be ascribed to the strong interaction among diverse elemental species of the sample.

Table 2 shows that after adding PTA, the peak area ratio of Ni^0^/Ni^2+^ in the NiMo/HZ0.5 and PTA20-NiMo/HZ0.5 catalysts increased from 30.5% to 43.3%, and the Mo^6+^/Mo^4+^ ratio increased from 60.0% to 71.5%. These changes suggested that the PTA-loaded catalyst was able to promote the reduction of Ni^2+^ and the oxidation of Mo^4+^. The Mo species may donate partial electrons to the Ni oxide species and keep metal oxide species in the reduction state. The electron transfer among metal species was due to the Keggin structure of PTA, which allowing replacement of the presulfurization and maintenance of the catalysts in the active form during the hydrocracking reaction^4^,^5^. The similar phenomenon was also reported in our previous work^5^,^6^.

The acidity of the ZSM-5 samples was studied by NH_3_-TPD. The acid properties of the different samples are summarized in Table 3. Two desorption peaks were observed at 220 °C and 380 °C approximately corresponding to the desorption of NH_3_. The adsorbed NH_3_ molecules desorbed from the weak acid sites at low temperatures, and from the strong acid sites at high temperatures. The weak acid sites were ascribed to the acidic framework of the Si–OH–Si group, whereas the strong acid sites were ascribed to the acidic framework of the Si–OH–Al group^34^. The loss of the weak and strong acid sites in the HZ0.5 and NiMo/HZ0.5 samples could be because desilication and dealumination reduced the amount of framework acid sites after alkali treatment and subsequent acid washing^15^. The other potential explanation for the reduction in acidity is that the acid sites located on the external/mesopore surface had lower acid strength than those inside the micropore system^43^. Overall, compared to the parent HZ catalyst, the hierarchical samples HZ0.5 and NiMo/HZ0.5 exhibited decreased total acidity, likely due to the partial loss of framework acid sites and the formation of mesopore structure. The incorporation of NiMo appeared to affect the number of weak acid sites. The increase in weak acid sites might be related to the formation of new sites by NiMo species with weak acidic features. For the hierarchical NiMo/HZ0.5 sample, the high temperature peak become broader and was shifted toward higher temperature (370 °C to 383 °C) compared to the NiMo-free hierarchical HZ0.5. These results may indicate the better dispersion and contact of the NiMo particles with the zeolitic support for the hierarchical zeolite samples, which should have a positive effect on the catalytic activity. The ratio of weak acid sites and strong acid sites was gradually increased for the catalysts. With the addition of PTA, the number of strong acid sites of PTA20-NiMo/HZ0.5 sample increased. This result suggested that the PTA was loaded on the zeolite surface, and the strong acidic protons of PTA caused the shift. Compared with the desorption temperature peak of NiMo/HZ0.5, the peak assigned to the strong acid sites were shifted to lower temperature, but the weak acid sites were shifted to higher temperature, showing moderation of the strength of the acid sites, which weakened the coke deposition and restrained the deactivation of the catalyst. The nature of a solid acid catalyst is mainly determined by the strong acid sites on the surface^44^.

### Analysis of the liquid products

**Figure 6:**
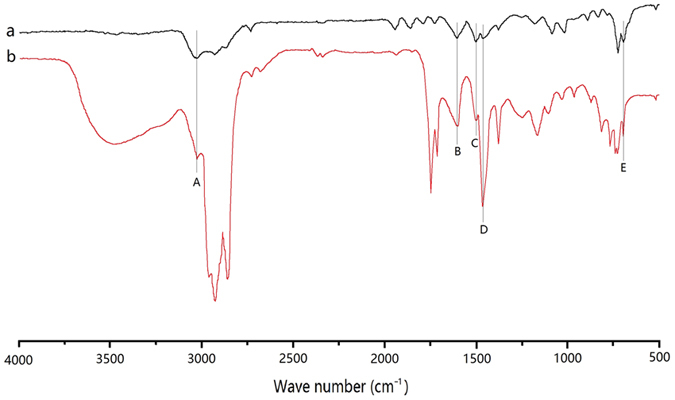
IR of (**a**) standard toluene and (**b**) liquid products produced using the HZ catalyst (A:CH_*x*_ stretching; BD: C=C stretching; C: C=C vibrations; E: aromatic rings vibrations).

**Figure 7:**
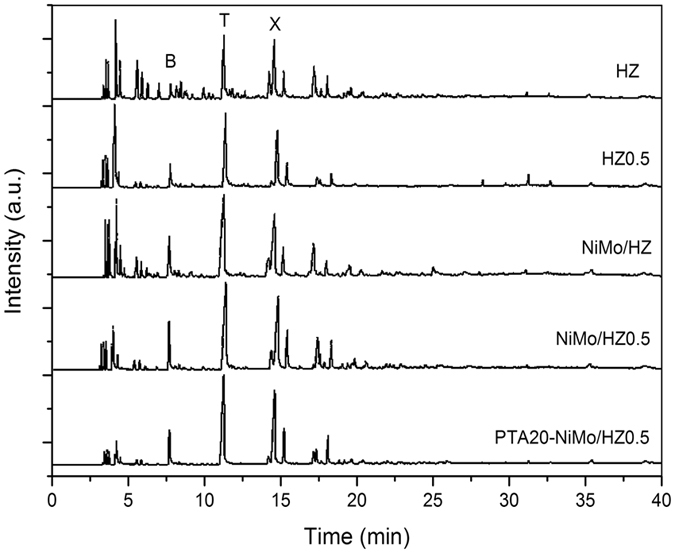
The GC analysis charts of liquid products.

**Figure 8:**
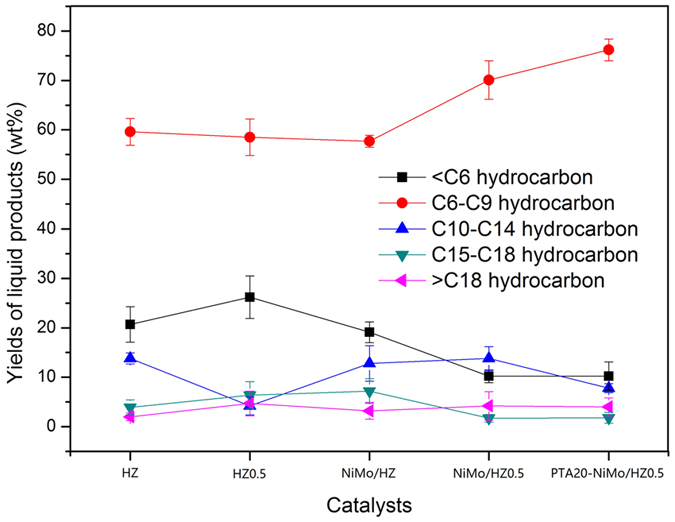
Hydrocarbon distribution of the liquid products analyzed by GC (wt%).

The FTIR spectra of the standard toluene and liquid products from the reaction using HZ catalyst are shown in Fig. 6. The GC analysis charts of liquid products over the catalyst are shown in Fig. 7. The hydrocarbon distribution of the products is shown in Fig. 8.

The structure was confirmed by FTIR spectra. The peaks at 2920 cm^−1^ and 2850 cm^−1^ were assigned to methylene groups^45^. These results were consistent with GC analysis of the liquid products produced using the HZ catalyst (Figs 7 and 8). The peak at 3050 cm^−1^ was due to aromatic CH_*x*_ stretching, the peak at 1600 cm^−1^ and 1421 cm^−1^ were attributed to the symmetric stretching of the C=C aromatic ring^45^,^46^,^47^, and the peak at 1500 cm^−1^ was due to aromatic C=C vibrations^48^,^49^. The peak at 752 cm^−1^ was assigned to the vibrations of aromatic rings with a low degree of substitution^45^. The characteristic peaks of the liquid products were consistent with the toluene standard, which suggested that aromatics were generated via the catalytic conversion of Jatropha oil.

**Figure 9:**
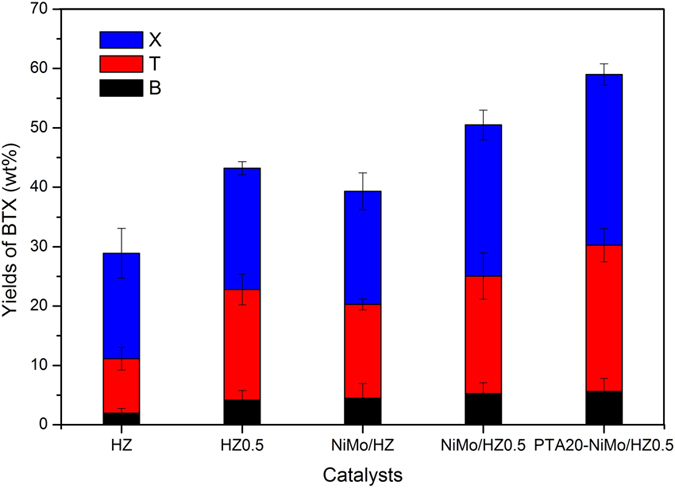
The yields of BTX aromatics in the liquid products.

The hydrocracking of Jatropha oil to bio-aromatics using the ZSM-5 catalysts was investigated. GC analysis charts of liquid products over the catalyst are shown in Fig. 7. The hydrocarbon distribution of the products is shown in Fig. 8. The amount of each product group was calculated based on the product of the relative area (%) of each component as determined by the GC analysis. The products consisted of three fractions, gaseous products, liquid products and coke. The liquid products accounted for about 70 wt% of the Jatropha oil, and the majority of the liquid products were BTX and light oil. The remaining 30 wt% was a gaseous product that was mainly composed of CO, CO_2_, CH_4_, C_2_H_4_, and C_3_H_8_. The C15–C18 fraction from the deoxygenation reactions of triglyceride was barely detected in the samples except for that prepared using the HZ0.5 catalyst sample that showed low amounts. The acidity of the catalysts may have been sufficient for the deoxygenation and cracking reactions, but the acidity of the HZ0.5 catalyst was slightly lower, according to the values presented in Table 3 24. In the cracking reaction, the C15–C18 fraction was cut off and broken into (C2–C10) fractions, which provided abundant materials for the aromatization^50^. The hydrocarbons in the liquid products were primarily hydrocarbon fractions of C6–C9 (Fig. 8). Analysis of the aromatic fraction indicated that it primarily consisted of the most valuable C6–C8 BTX aromatics (Fig. 9).

**Figure 10:**
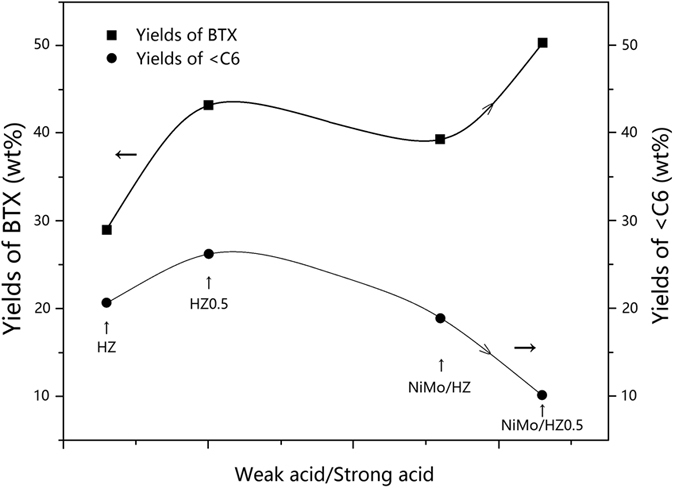
Correlation between the ratio of weak acid sites to strong acid sites and the yields of BTX or <C6 fractions for the different catalysts.

The <C6 light hydrocarbon fractions, including C2–C5 alkenes/alkanes, CO and CO_2_, might be direct materials for the aromatization, based on the correlation between the ratio of weak acid sites to strong acid sites and the yields of BTX or <C6 fractions (Fig. 10). The yield of BTX increased but the yield of <C6 fractions decreased, suggesting an inverse relationship. The yield of BTX aromatics was practically proportional to the ratio of weak acid sites and strong acid sites except for reactions performed using the PTA20-NiMo/HZ0.5 sample, which revealed that the weak acid sites of the catalyst might participate in the rate-limiting reaction for aromatization. For the PTA20-NiMo/HZ0.5 sample, although the weak and strong acid sites also increased, but the ratio of weak acid sites and strong acid sites decreased due to the more increment in strong acid sites.

**Figure 11:**
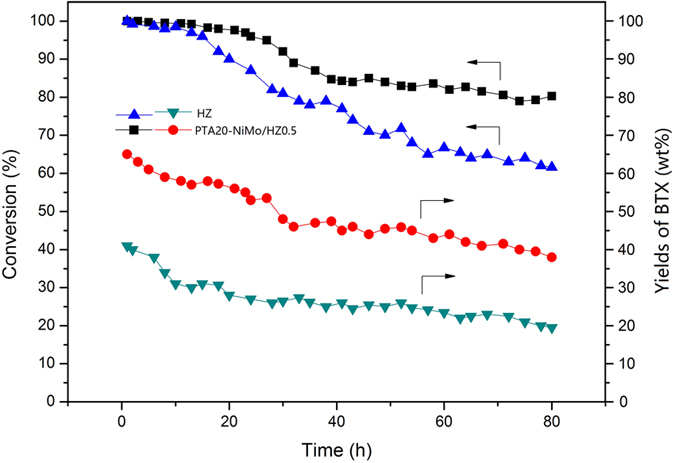
Conversion of Jatropha oil and BTX yields in product oil as a function of reaction time using the HZ and PTA20-NiMo/HZ0.5 catalysts.

The conversion of Jatropha oil and the yield of BTX using the catalysts (HZ, PTA20-NiMo/HZ0.5) are shown in Fig. 11. The conversion of Jatropha oil was more than 96.5% by 15 h for the two catalysts. The conversion gradually decreased from 98.3% to 84.7% between 15 h and 40 h for the PTA20-NiMo/HZ0.5 catalyst. For the HZ catalyst, the conversion quickly decreased from 96.5% to 68.1% between 15 h and 55 h. The conversions of the two catalysts exhibited little change after 55 h. The BTX yield was gradually decreased from 64.7% to 47.2% by 35 h and remained steady subsequently for the PTA20-NiMo/HZ0.5 catalyst. The BTX yields decreased from 41.6% to 26.5% by 30 h for the HZ catalyst. The results showed that the stability or catalytic activity of the prepared PTA20-NiMo/HZ0.5 catalyst were better than that of the HZ catalyst.

**Figure 12:**
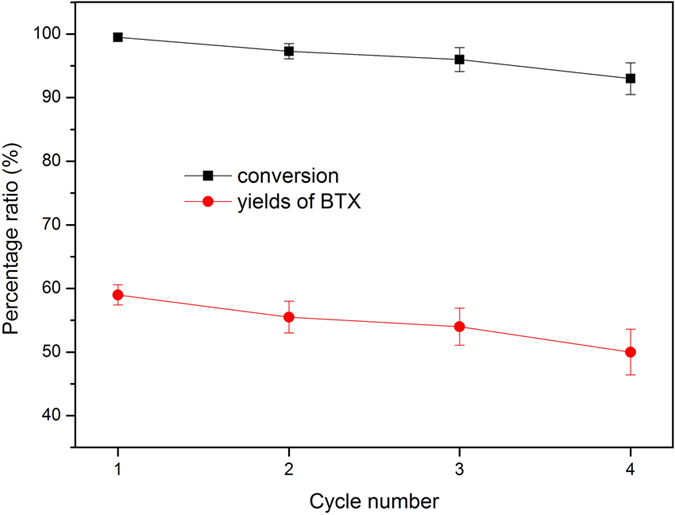
The conversion and yields of BTX for consecutive use of the PTA20-NiMo/HZ0.5 catalyst.

The stability and recyclability of the best-performing catalyst (PTA20-NiMo/HZ0.5) were assessed by performing four consecutive cycles. The conversion and yields of BTX over the catalyst (8 h) upon consecutive use are shown in Fig. 12. The catalyst was thermally treated in air to simulate a typical regeneration procedure between each cycle. The conversion gradually decreased from 99.5% to 93.2% and the BTX yields from 59.5% to 50.1%. Such a minor change just shown the stability of the catalyst activity.

**Figure 13:**
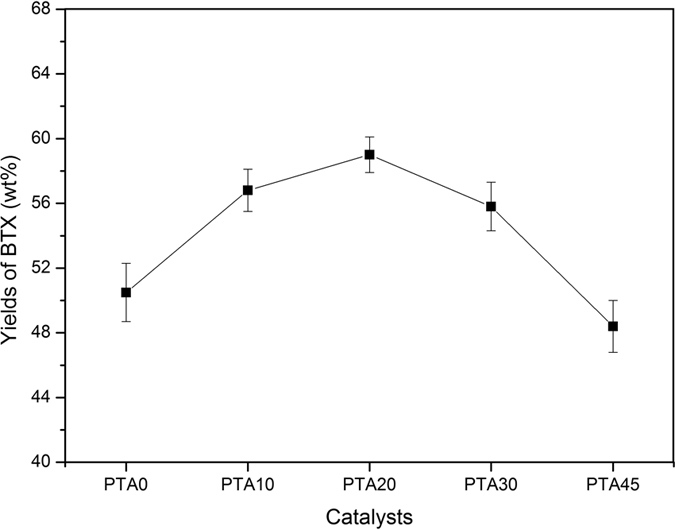
The yields of BTX aromatics for the NiMo/HZ0.5 catalysts (wt %) with various amounts of PTA.

A comparison of the BTX yields performance of the ZSM-5 revealed distinct aromatization activity for the different catalytic zeolites. For the HZ and NiMo/HZ catalysts, the latter sample exhibited higher formation of BTX aromatic products. The incorporation of NiMo metal promoted the formation of gaseous hydrocarbons likely associated with the occurrence of hydrogen transfer reactions, which increased the formation of light fractions and provided more materials for aromatization (Fig. 10). Despite the decreased acidic sites on the NiMo/HZ0.5 catalyst, the ratio of weak acid sites and strong acid sites was increased and the BTX aromatic products also increased compared to the NiMo/HZ sample. The difference in performance was only attributed to the hierarchical treatment. This may be because the hierarchical NiMo/HZ0.5 catalyst exhibited higher external and mesopore surface areas, bigger pore volume, and larger pore diameter (Table 1), which broadened the reaction area and improved the diffusion limitation. In addition, the active center located at the mesopore walls of the hierarchical zeolites could catalyze reactions involving bulky molecules that were unable to enter the zeolite micropores and enhanced the reaction accessibilities^51^. The NH_3_-TPD analysis indicated better dispersion and contact with NiMo in the NiMo/HZ0.5 catalyst which may act synergistically to increase activity. As listed in Fig. 13, all samples that contained PTA exhibited very high catalytic properties for the aromatization reaction. Moreover, the PTA20-NiMo/HZ0.5 catalyst exhibited the highest BTX yield (59 wt%) compared with the NiMo/HZ0.5 catalyst. This could be because there were more acid sites on the PTA20-NiMo/HZ0.5 catalyst than the NiMo/HZ0.5 catalyst. In addition, according to the XPS analysis, the Mo species might donate partial electrons to the Ni oxide species, resulting in the transfer of electrons between Mo and Ni, which could increase the production of carbenium ions and enhance catalytic activity.

### The possible reaction pathway

The majority of products converted from Jatropha oil were CO, CO_2_, CH_4_, C_2_H_4_, C_3_H_8_ and hydrocarbons ranging from C4 to C18. In the initial stage of the hydrocracking reaction, each triglyceride molecule was cracked into three hydrocarbon molecules via three types of deoxygenation reactions (equ. 3), which were hydrodecarboxylation (HDCO), hydrodecarbonylation (HDC) and hydrodeoxygenation (HDO)^52^. C15 and C17 hydrocarbons were the products of lesser hydrogen consumption, direct decarboxylation and decarbonylation (carbon removal pathway), and the C16 and C18 hydrocarbons were the products of the higher hydrogen consuming process of hydrodeoxygenation (hydrogen addition pathway).

Hydrocracking reaction:


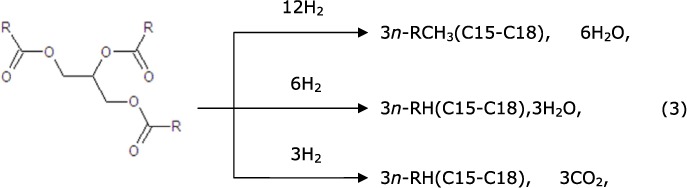


Meanwhile, the oxygen content of triglycerides was deleted as CO_2_, CO, and H_2_O. CO, CO_2_, CH_4_, C_2_H_4_, and C_3_H_8_ were the major gaseous products found in the effluent gas mixture. Methanation reactions (eqs 4 and 5) and water gas shift (equ. 7) were probable side-reactions, and these reactions can also increase hydrogen consumption or methane composition. The methane could be partly converted to ethylene (equ. 6), and might promote the methane dehydro-aromatization (MDA) reaction, which contributed to the BTX yields^53^,^54^. Two types of reactions that mainly utilized CO also occurred in the gaseous products. One was via the Fischer–Tropsch (equ. 8) reaction to generate alkylcarbenium ion. The other was via the Boudouard reaction (equ. 9) to result in the deposition of carbon on the surface of the catalyst^54^.

Methanation reaction:


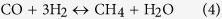



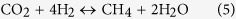



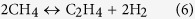


Water gas shift:


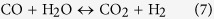


Fischer–Tropsch reaction (FT):


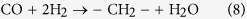


Boudouard reaction:


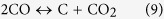


The subsequent cracking reaction might occur as follows^55^. A tiny fraction of the C15-C18 alkanes could be directly cracked (equ. 10) to generate the alkylcarbenium ion. A majority of the C15-C18 alkanes were dehydrogenated on the metal sites to generate the corresponding alkenes (equ. 12), then the alkenes moved to the strong acid sites and generated alkylcarbenium ion (equ. 13), and the alkenes were then rearranged and cracked via β-scission (eqs 11 and 13). Next, the generated light alkylcarbenium ion was oligomerized, and C2-C10 alkenes were generated (equ. 13) as the principal intermediate products of the aromatization reaction^50^,^56^. Finally, the alkenes moved to the metal sites and were hydrogenated to generate C2-C10 alkanes (equ. 12), and then the alkanes joined in the cracking circulation to generate lighter alkenes.

Subsequent cracking reaction:


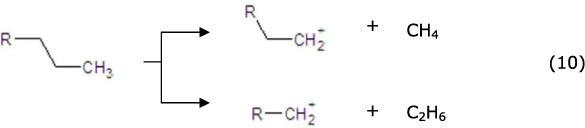



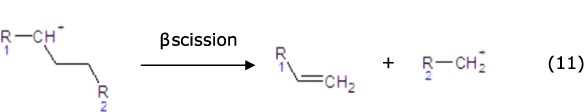


Dehydrogenation and rearranged reaction:


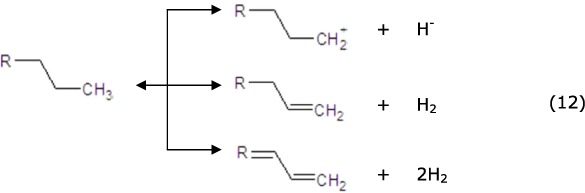



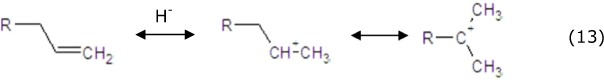


It was presumed that aromatics were formed by Diels–Alder reactions from light alkenes, which was the rate-limiting, key reaction for aromatization^56^. The formed alkenes came to the weak acid site to undergo the Diels–Alder cyclization reactions and produce the cycloalkanes, and then the cycloalkanes transformed into aromatics at strong acid sites by dehydrogenation-aromatization (eqs 14,15,16,17,18). Then, only a small portion of aromatics polymerized on the surface of the catalyst to form coke.

The Diels–Alder reactions and dehydrogenation-aromatization to form BTX:


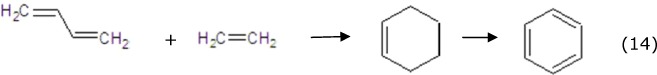



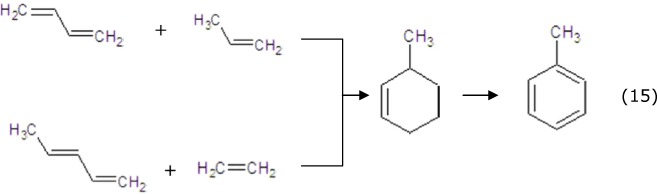



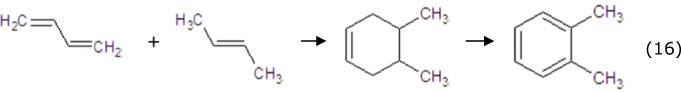



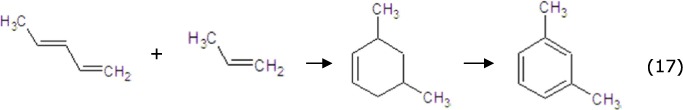



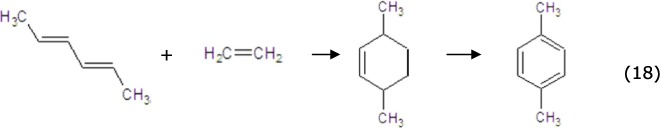


## Conclusions

This study presents the preparation of hierarchical PTA-NiMo/ZSM-5 catalysts with phosphotungstic acid (PTA) for the transformation of Jatropha oil into benzene, toluene, and xylenes (BTX) aromatics. The yield of BTX was 59 wt% at 380 °C, 3 MPa, H_2_/oil (v/v) = 1000, and LHSV = 1 h^−1^ for the reaction by the PTA20-NiMo/HZ0.5 catalyst (PTA 20 wt%). The optimal PTA amount was 20 wt%. The aromatization activity of the catalyst was enhanced by the moderate treatment of a hierarchical process, which was due to the increase in surface areas and improved diffusion/dispersion. The activity was further enhanced by the addition of PTA, because it increased the moderate acid sites or the electronic transformation to generate more carbenium ions to accelerate the reaction. Furthermore, the possible mechanism of BTX formation was presumed through Diels–Alder reactions and dehydrogenation-aromatization from the reaction pathway. This work presents a method to produce an efficient catalyst that does not require sulfurization, preventing environment and human health risks.

## Additional Information

**How to cite this article**: Yang, X. *et al*. Hydrocracking of Jatropha Oil over non-sulfided PTA-NiMo/ZSM-5 Catalyst. *Sci. Rep.* **7**, 41654; doi: 10.1038/srep41654 (2017).

**Publisher's note:** Springer Nature remains neutral with regard to jurisdictional claims in published maps and institutional affiliations.

## Figures and Tables

**Table 1:** Textural properties of the prepared ZSM-5 samples.

Sample	Si/Al (mol/mol)	Surface Area (m^2^ g^−1^)		Pore Volume (cm^3^ g^−1^)			Pore Diameter (nm)		
		S_BET_	S_meso_	S_micro_	V_total_	V_meso_	V_micro_	D_meso_	D_micro_
HZ	25	402	51	351	0.27	0.04	0.23	—	0.55
NiMo/HZ	25	346	35	311	0.23	0.03	0.20	—	0.51
NiMo/HZ0.2	23	393	127	266	0.39	0.21	0.18	3.56	0.52
NiMo/HZ0.5	22	458	235	223	0.42	0.26	0.16	5.67	0.50
NiMo/HZ1.0	19	264	172	92	0.26	0.15	0.11	6.75	0.51
PTA5-NiMo/HZ0.5	22	452	234	218	0.41	0.26	0.15	5.63	0.51
PTA20-NiMo/HZ0.5	22	447	232	215	0.40	0.25	0.15	5.61	0.50
PTA45-NiMo/HZ0.5	22	393	205	188	0.35	0.23	0.12	5.17	0.49

**Table 2:** Relative peak areas and ratios ofNi and Mo in the XPS analysis (%).

Catalyst	Ni			Mo		
	Ni^0^	Ni^2+^	Ni^0^/Ni^2+^	Mo^4+^	Mo^6+^	Mo^6+^/Mo^4+^
NiMo/HZ0.5	23.4	76.6	30.5	62.5	37.5	60.0
PTA20-NiMo/HZ0.5	30.2	69.8	43.3	58.3	41.7	71.5

**Table 3:** The acidity the catalysts (mmolg^−1^).

Sample	The strength of the acid sites				Total	Weak/Strong
	Weak		Strong			
	Peak	Acidity	Peak	Acidity		
	Temp.(°C)	(100–300 °C)	Temp.(°C)	(300–600 °C)		
HZ	215	0.35	392	0.31	0.66	1.12
HZ0.5	218	0.29	370	0.24	0.53	1.21
NiMo/HZ	217	0.41	392	0.30	0.71	1.36
NiMo/HZ0.5	210	0.33	383	0.23	0.56	1.43
PTA20-NiMo/HZ0.5	230	0.38	377	0.55	0.93	0.69
